# Metabolic profiling of serum and tissue from the rotator interval and anterior capsule in shoulder stiffness: a preliminary study

**DOI:** 10.1186/s12891-019-2709-7

**Published:** 2019-08-07

**Authors:** Hyo-Jin Lee, Oak-Kee Hong, Dong-Ho Kwak, Yang-Soo Kim

**Affiliations:** Department of Orthopedic Surgery, Seoul St. Mary’s Hospital, The Catholic University of Korea, 505 Banpo-dong, Seocho-gu, Seoul, 137-701 South Korea

**Keywords:** Shoulder, Shoulder stiffness, Capsule, Rotator interval, Metabolic profile, Sphingomyelin, Glycerophospholipid

## Abstract

**Background:**

Using mass spectrometry, we evaluated the metabolic profiles of patients who had rotator cuff tears with shoulder stiffness, or shoulder stiffness only, and compared these with samples from a control group.

**Methods:**

This study enrolled 28 patients, including 10 patients with shoulder stiffness only (group I), nine patients with rotator cuff tear and stiffness (group II), and nine controls selected from patients diagnosed with impingement syndrome or long head of the biceps lesions without evident limitation of joint motion or rotator cuff tears. Serum and tissue from the rotator interval and anterior capsule were collected. In all, 82 samples were analyzed for metabolite profiling using the AbsoluteIDQ™p180 Kit.

**Results:**

Comparison of 186 metabolites revealed that groups I and II had significantly higher concentrations of sphingolipids in serum (SM C24:1; group I = 65.16 μm, group II = 68.07 μm) than controls (55.37 μm, *p* = 0.005 & 0.015, respectively). Higher concentrations of sphingolipids were also present in the rotator interval tissue (SM C22:3) of groups 1 (0.0197 μm) and 2 (0.0144 μm) than controls (0.0081 μm, *p* = 0.012 & 0.014, respectively). The concentration of glycerophospholipid (PC aa C30:0) was higher in the anterior capsule tissue of groups I (0.850 μm) and II (1.164 μm) than controls (0.572 μm; *p* = 0.007) Total cholesterol was positively correlated with sphingolipid concentration in serum (SM C24:1, rho = 0.782, *p* = 0.008) and rotator interval tissue (SM C22:3, rho = 0.750, *p* = 0.017). There was no significant difference in the metabolites evaluated in groups I and II.

**Conclusion:**

Metabolic profiling showed that levels of lipid-related metabolites were increased in the anterior capsule tissue and rotator interval tissue of patients with shoulder stiffness. Sphingomyelin (SM C22:3) in the tissue of the rotator interval was positively correlated with the serum level of total cholesterol in patients with shoulder stiffness only. The level of glycerophospholipid (PC30:0) in the anterior capsule was positively correlated with the serum level of total cholesterol in patients who had rotator cuff tear with shoulder stiffness. The results indicate that serum total cholesterol may be related to shoulder stiffness. Future studies are needed to evaluate the role of serum cholesterol in the pathogenesis of shoulder stiffness.

**Trial registration:**

KC12OISI0532. Registered Nov 15, 2012. approval by the Institutional Review Board of Seoul St. Mary’s Hospital, the Catholic University of Korea.

## Background

Shoulder stiffness is a common pathological condition that manifests as restriction in active and passive range of motion. Shoulder stiffness involves both inflammatory and fibrotic processes [1, 2]. The pathophysiology includes synovial inflammation, subsynovial fibrosis, and subsequent capsular fibrosis and thickening, collectively leading to glenohumeral joint contracture [1]. There are many known systemic risk factors that can lead to the biological and histological changes characteristic of shoulder stiffness [2–9]. A recent study reported that hypercholesterolemia and inflammatory lipoproteinemias are strongly associated with primary adhesive capsulitis [10]. Although shoulder stiffness is a consequence of local inflammatory and fibrotic change in the capsule tissue, an increased concentration of soluble intercellular adhesion molecule-1 (ICAM-1) in serum of patients with adhesive capsulitis or diabetes mellitus is a good example of the connection with systemic conditions [11]. Nevertheless, the relationships between certain systemic diseases and shoulder stiffness and the underlying pathological mechanism have not been clearly determined. Besides primary or idiopathic causes, shoulder stiffness frequently accompanies a rotator cuff tear [12]. As there are various external and internal conditions that lead to shoulder stiffness, it is important to identify common factors involved with shoulder stiffness. Doing so requires an inspection tool that can simultaneously screen and evaluate related factors among a huge number of candidates.

Metabolic profiling is a technique for identifying and quantifying metabolites [13]. Changes in metabolites in body fluids and tissues can be direct indicators of changes in physiology and pathology [14]. We were interested in assessing the insights that metabolic profiling could provide into shoulder stiffness, as this condition is not only confined to local reactions within the shoulder, but is also involved in several systemic diseases and conditions. To our knowledge, metabolomics has not yet been applied to study stiffness of the shoulder. The purpose of this study was to investigate and evaluate if certain metabolites are related to the pathogenesis of shoulder stiffness. We used a targeted metabolic profiling technique to identify metabolites from blood (serum) and tissues from the rotator interval and anterior capsule in patients with shoulder stiffness. First, we quantitatively compared the expression of metabolic profiles among patients with primary shoulder stiffness, rotator cuff tear with shoulder stiffness, and a control group. Then, we investigated whether specific metabolic biomarkers were associated with the serum level of total cholesterol and fasting glucose, as total cholesterol and fasting glucose are known to be related to the etiology of shoulder stiffness. Our hypothesis was that metabolites associated with lipid and glucose metabolism are positive correlated with the incidence of shoulder stiffness.

## Material and methods

From October 2013 to December 2014, we enrolled a total of 28 consecutive patients. We defined shoulder stiffness as forward flexion of less than 100°, external rotation less than 45° (maximal 90°), or internal rotation of the back at a level lower than the first lumbar spine junction (maximal T7 level) in passive range of motion [15]. Magnetic resonance imaging (MRI) was performed to confirm accompanying diseases.

Patients assigned to group I were required to have only primary shoulder stiffness without any pathology in the rotator cuff tendons or within the glenohumeral joint or signs of acromio-clavicular and/or glenohumeral osteoarthritis. Prior to arthroscopic surgery (capsulectomy/capsular release), patients had undergone at least 3 months of non-operative treatment for shoulder stiffness but showed no remarkable improvement. Patients assigned to group II were required to have a small or medium-sized (tear size < 2 cm) full-thickness rotator cuff tear confirmed by MRI before surgery along with concomitant shoulder stiffness. The same definition of shoulder stiffness was used for groups I and II. For the control group, tissues were acquired from patients diagnosed with impingement syndrome or long head of the biceps lesions without evident limitation of joint motion or rotator cuff tears. The demographic data of the enrolled patients are summarized in Table 1. This study was approved by the Institutional Review Board of our hospital, and all patients provided written informed consent. Written form of Informed consent was obtained from all participants.

**Table 1:** Demographic Data

	Control	Group I (primary shoulder stiffness)	Group II (rotator cuff tear with shoulder stiffness)
No. of patients, n	9	10	9
Age, mean (range), years	57.3	57.5	59.5
Sex, male/female, n	3/6	3/7	3/6
Patients with diabetes mellitus, n	1	2	2
Average serum level of total cholesterol (mg/dl)	189.03 (±7.04)	171.5 (±8.75)	183.2 (±9.47)
Average serum level of fasting glucose (mg/dl)	114.42 (±14.28)	126.9 (±5.18)	117.2 (±9.16)

### Metabolomic analysis

Tissues from the rotator interval and anterior capsule were collected during arthroscopic surgery. Peripheral whole blood samples (20 cc) were collected from a vein into Vacutainer tubes after a 12-h overnight fast before surgery. Within 30 min, plasma was separated from whole blood using a standard protocol and stored at − 80 °C. Tissues from the rotator interval and anterior capsule were processed based on the instructions provided with the metabolomics assay kit.

### Targeted profiling analysis of tissue and plasma

Twenty-eight serum samples, 28 rotator interval tissue samples, and 26 anterior capsule tissue samples were collected from subjects in the three groups. These 82 samples were analyzed with the Absolute IDQ™ p180 Kit (BIOCRATES Life Science AG, Innsbruck, Austria). Amino acids and biogenic amines were analyzed quantitatively by stable isotope dilution in LC-MS/MS using an API 4000Qtrap (ABSCIEX, Foster City, CA, USA) mass spectrometer. For acylcarnitines, hexoses, phosphor-and sphingolipids, mass spectrometry analysis was done by the FIA-MS/MS method. Quantification of metabolite concentrations and automatic quality assessment were performed by the MetVal™ module (BIOCRATES Life Science AG, Innsbruck, Austria). Concentrations of all analyzed metabolites are reported in units of μM/L.

### Data process and statistical analysis

The Absolute IDQ™ p180 kit was used to analyze 186 metabolites in 28 samples of serum and 54 samples of tissue. Differences in levels of all 186 metabolites among the disease and control groups were evaluated. Statistical analyses were performed with SPSS software (version 18.0; SPSS Inc., Chicago, IL, USA). Due to non-normal distributions of samples, non-parametric methods were used for the statistics. The Kruskal-Wallis test was used to compare differences in levels of metabolites among groups. The level of statistical significance was set at *p* < 0.017 (5%/3) by applying Bonferroni correction to address the problem of multiple comparisons for post hoc analysis. For those metabolites that were significantly different among groups, Spearman rank correlation coefficients (ρ) were used to determine the relationships among the levels of the selected metabolites and serum total cholesterol and fasting glucose levels. The level of statistical significance was set at *p* < 0.05.

## Results

### Comparison of the metabolite profiles of the control and disease groups

A total of 82 samples were collected for the study. Only levels of metabolites with values above the limit of detection (LOD) were considered. Among the 186 metabolites measured in serum, rotator interval tissue, and anterior capsule tissue, 83 metabolites below LOD were excluded. From the remaining 103 metabolites, four metabolites showed significant differences among groups.

**Fig. 1:**
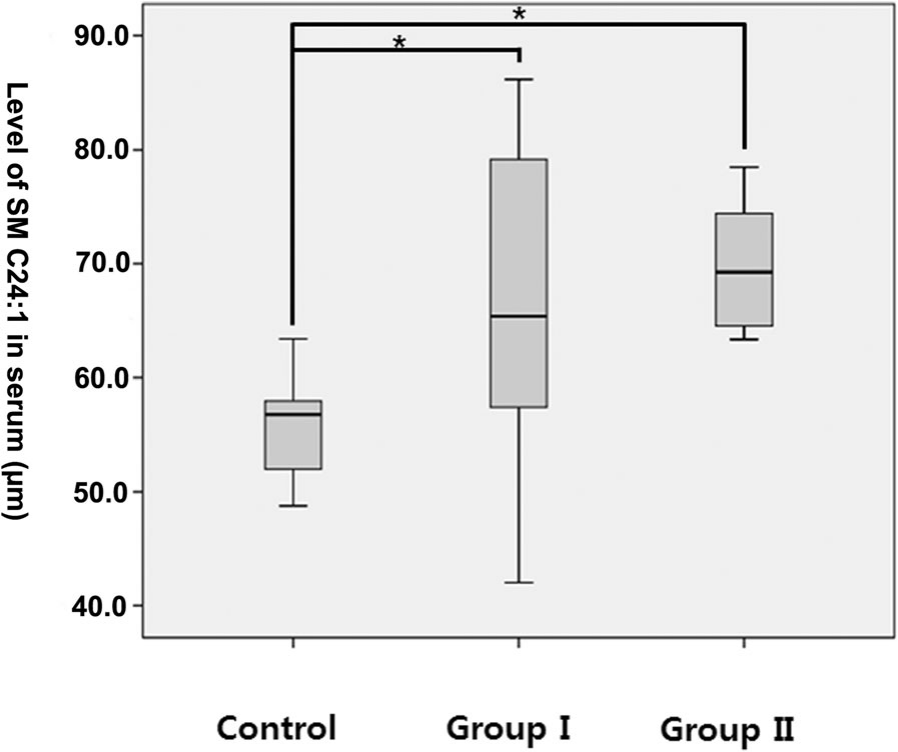
Comparison of SM C24:1 in serum. *p = 0.005 & 0.015

**Fig. 2:**
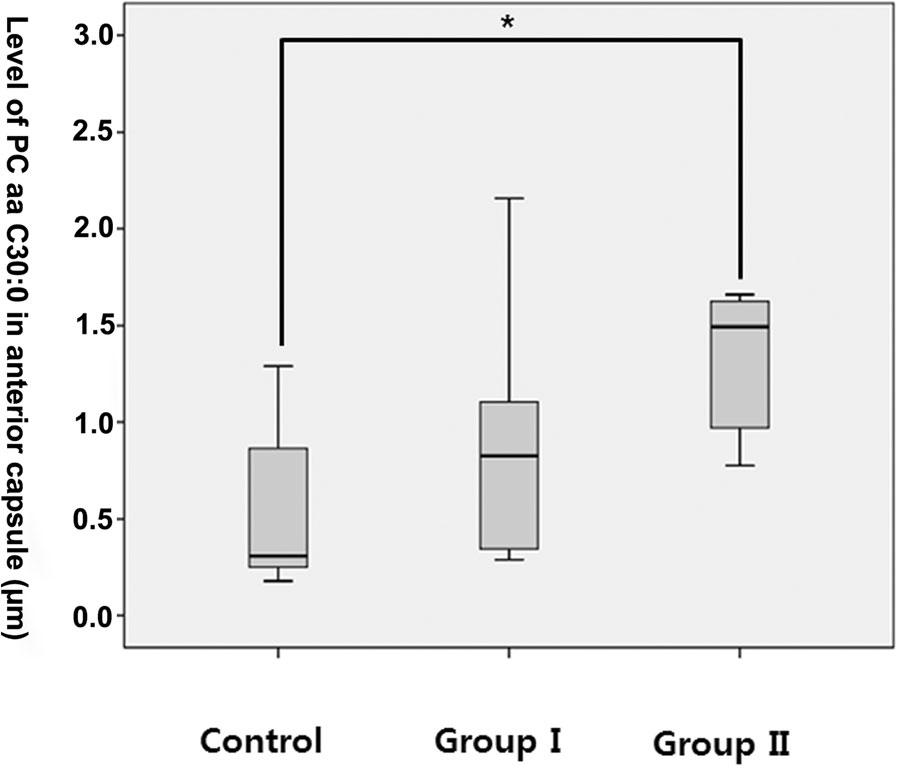
Comparison of PC aa C30:0 in anterior capsule tissue. *p = 0.007

**Fig. 3:**
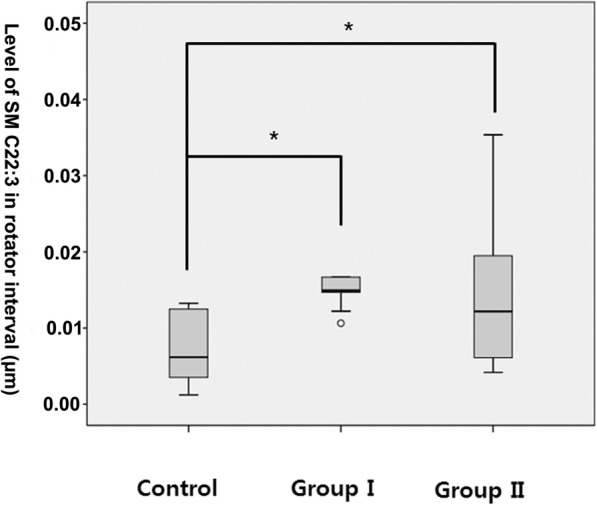
Comparison of SM C22:3 in rotator interval tissue. *p = 0.012 & 0.014

Significant increases in metabolites from lipid classes, such as sphingomyelin and glycerophospholipid, were observed in the diseased versus control biological materials. In the serum analysis, groups with shoulder stiffness (groups I and II) had significantly higher levels of sphingomyelin (SM C24:1) (group I: 65.16 ± 13.09 μm, group II: 68.07 ± 8.22 μm) than the control group (55.37 ± 4.41 μm) (*p* = 0.005 & 0.015, respectively) (Fig. 1). There was no significant difference between groups I and II. Glycerophospholipid (PC aa C30:0) was significantly higher in the anterior capsule samples of group II (1.15 ± 0.76 μm) than the control group (0.572 ± 0.477 μm) (*p* = 0.007) (Fig. 2). Sphingomyelin (SM C22:3) was significantly higher in the rotator interval tissue of groups I (0.0197 ± 0.0108 μm) and II (0.0144 ± 0.0098 μm) than in the control group (0.0081 ± 0.0081 μm) (*p* = 0.012 & 0.014, respectively) (Fig. 3). However, there were no significant differences in metabolites in the anterior capsule and rotator interval tissues between groups I and II.

### Correlations between metabolites, serum cholesterol, and fasting glucose level

In group I, the level of serum sphingomyelin (SM C24:1) was positively correlated with the level of total cholesterol (ρ = 0.782, *p* = 0.008) (Table 2). Glycerophospholipid (PC aa C30:0) derived from the anterior capsule showed a positive correlation with total cholesterol in group II (ρ = 0.683, *p* = 0.042) (Table 3). Sphingomyelin (SM C22:3) derived from rotator interval tissue showed a positive correlation with total cholesterol in group I (ρ = 0.750, *p* = 0.017) (Table 4). We did not find any correlation between the metabolites and serum glucose level in this study.

**Table 2:** Associations of level of serum SM C24:1 (sphingolipids) with total cholesterol and fasting blood sugar

	Control		Group I		Group II	
	rho	*P* value	rho	*P* value	rho	*P* value
Total Cholesterol	0.243	0.529	0.782	0.008	0.083	0.831
Fasting Blood Sugar	−0.033	0.932	−0.587	0.074	0.183	0.637

**Table 3:** Associations of level of anterior capsule PC aa C30:0(glycerophospholipid) with total cholesterol and fasting blood sugar

	Control		Group I		Group II	
	rho	*P* value	rho	*P* value	rho	*P* value
Total Cholesterol	0.342	0.452	0.224	0.533	0.683	0.042
Fasting Blood Sugar	−0.126	0.788	0.208	0.564	−0.183	0.637

**Table 4:** Associations of level of rotator interval SM C22:3(sphingomyelin) with total cholesterol and fasting blood sugar

	Control		Group I		Group II	
	rho	*P* value	rho	*P* value	rho	*P* value
Total Cholesterol	0.168	0.691	0.750	0.013	0.167	0.693
Fasting Blood Sugar	0.036	0.933	−0.633	0.067	0.143	0.736

## Discussion

This study is the first to investigate the metabolic features of shoulder stiffness. Using a targeted metabolic profiling platform, we successfully identified alterations in sphingomyelin and glycerophospholipid from serum, anterior capsule tissue, and rotator interval tissue in patients with shoulder stiffness when compared with a normal control group.

Sphingomyelin and glycerophospholipid, which were present at significantly higher levels in patients with shoulder stiffness, are major components of cell membranes and are related to lipid metabolism. Sphingomyelin is a type of sphingolipid found in animal cell membranes that contains phosphocholine or phosphoethanolamine as a polar group, and it is classified as a phospholipid. Phospholipid groups are important structural components of plasma lipoproteins and cell membranes that play a role in regulation of cell function, trafficking of membrane protein, and inflammation [16]. Sphingomyelin has a strong affinity for cholesterol and is correlated with the quantity of cholesterol in cell membranes. It plays an important role in metabolism of lipoproteins in plasma, including absorption and efflux of cholesterol, synthesis of bile acids, cholesterol esters, and other metabolites [17–19]. Increased plasma sphingomyelin levels have been reported to be clinically involved with atherosclerosis [20], coronary heart disease [21], obesity [22], and diabetes mellitus [23]. Phosphatidylcholines are glycerol-based phospholipids and are a major component of biological membranes.

Many previous studies have reported a relationship between abnormalities in serum lipid profiles and the incidence of adhesive capsulitis. Similar to Dupuytren contracture, hyperlipidemia is considered a possible risk factor for adhesive capsulitis [2]. Furthermore, a previous study reported higher levels of serum cholesterol and triglycerides in patients with adhesive capsulitis than in the normal control group [24]. Hand et al. designated hypercholesterolemia as a risk factor for adhesive capsulitis because it showed a prevalence of 17% among patients with adhesive capsulitis [25]. Park et al. recently reported that hypercholesterolemia and inflammatory lipoproteinemias have significant associations with primary adhesive capsulitis [10]. They proposed that higher levels of inflammatory lipoproteins in shoulder stiffness may induce inflammatory changes that are involved in adhesive capsulitis.

There are many lines of evidence that sphingomyelin, which is an integral part of various lipid membranes present in cells and organelles, is influenced by impaired lipid metabolism. In a rodent study, sphingomyelin was modestly but significantly increased in mice fed a high-fat diet [26]. Patients with breast cancer, who have elevated levels of plasma (or serum) lipids, also have higher levels of sphingomyelins in plasma samples than healthy controls [27]. We found that the sphingomyelin (SM C22:3) level in the tissue of the rotator interval was positively correlated with serum total cholesterol level in patients with primary shoulder stiffness (group I). Serum sphingomyelin (SM C24:1) in group I also showed a positive correlation with serum total cholesterol. Furthermore, the level of glycerophospholipid (PC30:0) in the anterior capsule showed a positive correlation with serum total cholesterol level in patients having rotator cuff tears with shoulder stiffness (group II). These findings suggest that the serum total cholesterol level may be involved in the pathogenesis of shoulder stiffness. However, further studies are required to determine how changes in lipid profile affect the metabolic condition of tissues in shoulder stiffness. It remains unclear whether disruption of lipid metabolism is a trigger for shoulder stiffness or whether the condition itself produces an increased concentration of lipid metabolites.

In contrast to our finding of differences in lipid profiles among groups, we did not detect metabolic changes related to blood sugar level in patients with shoulder stiffness. A continuously increased glucose level, as in diabetes mellitus, is a well-known risk factor for frozen shoulder [4, 28, 29]. However, the three metabolites that showed a significant increase in the groups with shoulder stiffness (groups I and II) did not show any significant correlation with serum glucose level. Among the 28 patients enrolled, five patients (group I: *n* = 2, group II: *n* = 2, control: *n* = 1) were diagnosed with diabetes mellitus under control of medications, while only one patient from group II had an abnormal blood glucose level (> 126 mg%). The small number of patients with diabetes in our study may explain why we did not find any associations between metabolites and blood glucose levels. Although we could not find any direct correlations between certain metabolites and the serum level of blood glucose, SM C24:1 and SM C22:3 are known to be positively associated with insulin secretion and glucose tolerance [30].

This experiment was a preliminary study, and it had the advantage of applying metabolic profiling to evaluate the etiology of shoulder stiffness for the first time. Most previous studies have concentrated on evaluating histological and genetic features of adhesive capsulitis of the shoulder. Although this study was a pilot experiment, the association between metabolites related to lipid metabolism and lipid profile, which is known to be associated with shoulder stiffness, cannot be ignored. Metabolic profiling identifies and quantifies low-molecular weight compounds that are intermediates or endpoints of metabolism. As metabolites change rapidly in response to physiologic perturbations, they may be more proximal indicators of intermediary or disease phenotypes than proteins.

This study had some limitations. First, a proper sample size calculation was not performed, and the number of individuals per group was small. Due to the limited samples allowed per plate (82 samples per plate), the sample composition of each group was less than 10. As a result, we could use non-parametric statistical tests only. The small sample size also increased the likelihood of type II error. Second, the composition of samples was inhomogeneous (mixture of serum and tissue samples). Most previous metabolomic studies have evaluated samples in fluid form, such as synovial fluid, plasma, serum, or cerebrospinal fluid. Although there are no particular limitations regarding the sample type specified in the kit manual, more research is required to confirm that the results are consistent among different sample types. Third, this study could have been improved by taking consideration of multiple variables, including genetic predisposition to diabetes, basal blood sugar levels, age, history of other clinical conditions, medications, levels of physical exercise or activity.

Despite the small number of metabolites that were significantly associated with lipid metabolism, we do not believe it is coincidence that that these relationships were found. Further study is expected to identify the mechanism explaining how certain metabolites involved with the lipid profile participate in the pathogenesis of shoulder stiffness.

## Conclusions

Metabolic profiling showed that levels of lipid-related metabolites (sphingomyelin and glycerophospholipid) were increased in the anterior capsule and rotator interval tissues of patients with stiffness. Furthermore, sphingomyelin (SM C22:3) in the tissue of the rotator interval showed a positive correlation with serum total cholesterol level in patients with stiffness only. Glycerophospholipid (PC30:0) of the anterior capsule showed a positive correlation with serum total cholesterol level in patients with rotator cuff tear with stiffness. The results indicate that the serum total cholesterol level may be related to shoulder stiffness. Future studies are needed to evaluate the role of serum cholesterol in the pathogenesis of shoulder stiffness.

## Data Availability

The datasets used and/or analyzed during the current study are available from the corresponding author on reasonable request.

## Abbreviations

PC: Phosphatidylcholine
SM: Sphingomyelin
